# Association between care by hypertension specialists and major adverse cardiovascular events in patients with uncontrolled hypertension

**DOI:** 10.3389/fcvm.2026.1697092

**Published:** 2026-02-09

**Authors:** Li Cai, Ling Yao, Qing Zhu, Sha-sha Liu, Duo-lao Wang, Jing Hong, Mulalibieke Heizhati, Nan-fang Li, the Hypertension Specialists Medical Care Study Research Group, De-lian Zhang, Qin Luo, Meng-hui Wang, Jun-li Hu, Guo-liang Wang, Ying-chun Wang, Nuerguli Maimaiti, Ke-ming Zhou, Lei Wang, Xiao-guang Yao, Wen Jiang, Le Sun

**Affiliations:** 1 Hypertension Center of People’s Hospital of Xinjiang Uygur Autonomous Region, Urumqi, China; 2 Xinjiang Hypertension Institute, Urumqi, China; 3 NHC Key Laboratory of Hypertension Clinical Research, Urumqi, China; 4 Key Laboratory of Xinjiang Uygur Autonomous Region “Hypertension Research Laboratory”, Urumqi, China; 5 Xinjiang Clinical Medical Research Center for Hypertension (Cardio-Cerebrovascular) Diseases, Urumqi, China; 6 Department of Clinical Sciences Liverpool School of Tropical Medicine, Liverpool, United Kingdom

**Keywords:** a retrospective cohort, hypertension specialists, major adverse cardiovascular events, medical care, uncontrolled hypertension

## Abstract

**Background:**

The impact of medical care by hypertension specialists on the risk of subsequent major adverse cardiovascular events (MACE) among uncontrolled hypertensive patients remains unclear. We aimed to investigate the association between care by hypertension specialists and the risk of MACE among patients with uncontrolled hypertension.

**Methods:**

Using the Urumqi Hypertension Database (UHDATA), we studied a retrospective cohort of patients aged 45–79 years who were admitted for uncontrolled hypertension at People's Hospital of Xinjiang Uygur Autonomous Region, China, between 2015 and 2019. Based on hospitalization departments, we identified patients who had been exposed to medical care by hypertension specialists at least once and divided patients into a hypertension specialists group and a non-specialists group. Cox proportional hazards modeling was used to estimate the risk for MACE (a four-component outcome of cardiovascular death, non-fatal stroke, non-fatal myocardial infarction, and coronary revascularization) in the cohort using the propensity score method of stabilized inverse probability of treatment weighting (sIPTW).

**Results:**

A total of 10,680 patients with uncontrolled hypertension were analyzed, with a median follow-up of 4.0 years. Of these, 5,646 (52.9%) patients received medical care by hypertension specialists and experienced fewer MACE than the non-specialists group [21.5 vs. 39.7 per 1,000-person-year, adjusted hazard ratio (HR) 0.67, 95% confidence interval (CI) 0.57–0.79] after sIPTW. Results persisted for the MACE component, non-fatal stroke (HR 0.62, 95% CI 0.49–0.79), non-fatal myocardial infarction (HR 0.48, 95% CI 0.33–0.69), and coronary revascularization (HR 0.71, 95% CI 0.55–0.93). In subgroup analyses, no significant interaction effect was observed between medical care by hypertension specialists and key subgroup factors on MACE.

**Conclusions:**

This study demonstrated a significant association between medical care by hypertension specialists and a reduced risk of MACE in patients with uncontrolled hypertension. Our results suggest that medical care by hypertension specialists may play an essential role in improving cardiovascular outcomes among this high cardiovascular disease risk population.

## 1 Introduction

Hypertension is the leading global modifiable risk factor for cardiovascular disease (CVD) and premature death (1, 2). Uncontrolled blood pressure (BP) is associated with adverse cardiovascular outcomes (3, 4). Although pharmaceutical treatment and lifestyle modifications have demonstrated effectiveness as blood pressure interventions in numerous randomized clinical trials (5, 6), the rate of uncontrolled hypertension remains high at approximately 80% worldwide (7, 8). Furthermore, CVD incidence demonstrates a consistent age-dependent upward trend, with a particularly striking elevation among adults aged 45 years and older (9). The large population with uncontrolled hypertension is a major contributor to the continuing increase in cardiovascular disease burden (10), underscoring that a substantial number of cardiovascular events are preventable (11).

Previous studies have demonstrated that a physician's specialty impacts patient outcomes in cardiovascular disease (12–14). Several observational studies have indicated hypertension specialists provide advantages in controlling blood pressure and managing other cardiovascular risk factors (15–17). Hypertension specialists possess advanced expertise in the management of hypertension and play an essential role in evaluating and managing secondary causes of hypertension, as well as optimizing treatment based on customary care practices (18). However, the impact of care provided by hypertension specialists for patients with uncontrolled hypertension remains uncertain, and the long-term evidence regarding the risk of cardiovascular events is still lacking. This study employed a retrospective cohort of patients with uncontrolled hypertension to examine the association between care provided by hypertension specialists and the risk of subsequent major adverse cardiovascular events (MACE) in this population.

## 2 Materials and methods

### 2.1 Study design and participants

We conducted a retrospective cohort study by evaluating electronic medical records data of hypertensive patients who were admitted to the non-surgical department for hypertension at People's Hospital of Xinjiang Uygur Autonomous Region between January 2015 and December 2019, utilizing the Urumqi Hypertension Database (UHDATA) (19). Comprehensive details of this database have been published and are summarized in the online Supplementary Material (Supplementary Methods). All participants in this study were residents of Urumqi, with equitable access to the hospital's outpatient services. Patients attended the internal medicine clinic based on personal healthcare needs or preferences, or were referred from primary care facilities for uncontrolled or complex hypertension. The inclusion criteria were as follows: (1) patients aged 45–79 years and resident in Urumqi (*n* = 19,722); (2) patients with uncontrolled hypertension (*n* = 13,888), defined as having a mean systolic/diastolic BP of ≥140/90 mmHg, or ≥130/80 mmHg if they have existing CVD, diabetes mellitus (DM), chronic kidney disease (CKD), or a high risk of developing CVD (20, 21). Blood pressure was recorded in the research department as the average of three consecutive automated readings on the left arm at 30-s intervals, taken after ≥5 min of seated rest with back support, feet flat, and the arm positioned at heart level. The exclusion criteria included a prior history of myocardial infarction (*n* = 487), coronary revascularization (*n* = 539), or stroke (*n* = 669) at baseline, as well as no record of follow-up data (*n* = 1,513).

This investigation was performed in compliance with the Declaration of Helsinki and received approval from the hospital’s ethics committee (ethical approval NO. KY2022080904). Given the study's retrospective nature, no additional informed consent was required.

### 2.2 Data collection

Baseline information for all participants included the following variable: sociodemographic data encompassed age, sex, marital status, occupation, cigarette use, and alcohol consumption; anthropometric measurements comprised body mass index (BMI, calculated by the weight in kilograms divided by the square of height in meters), systolic blood pressure, and diastolic blood pressure; medical history included comorbidities such as ischemic heart disease (IHD, ICD-10:I20-I25), chronic cerebral hypoperfusion (CCH, ICD-10:I67.8), CKDs (ICD-10:N18), DM (ICD-10:E10-E11), and duration of hypertension, as well as antihypertensive therapy; and medical visit records included all visit types, inpatient admission and discharge departments, the presence of consultations or referrals, and all disease diagnoses and corresponding ICD-10 codes. Peripheral venous blood was collected at baseline following a 12-h fast for biochemical testing, which included serum triglycerides (TG), total cholesterol (TC), low-density lipoprotein cholesterol (LDL-c), high-density lipoprotein cholesterol (HDL-c), fasting glucose, uric acid (UA), serum creatinine, and the estimated glomerular filtration rate (eGFR, calculated using the 2021 Chronic Kidney Disease Epidemiology Collaboration creatinine equation: eGFR = 142*[min (Scr/0.7, 1) ^(−0.241)]*[max (Scr/0.7, 1)^(−1.200)]*(0.9938^age) *1.012[if female]; eGFR = 142* [min (Scr/0.9, 1) ^(−0.302)]*[max (Scr/0.9, 1) ^(−1.200)] *(0.9938^age)[if male]).

### 2.3 Exposure and groups

The exposure was care provided by hypertension specialists at the Hypertension Center of the hospital. This care involved guideline-based, systematic management procedures in addition to usual care. Its core components included (1) a comprehensive assessment of blood pressure control, overall cardiovascular risk, and hypertensive complications; (2) timely screening and identification of secondary causes of hypertension; and (3) integrating etiology treatment into management strategies, and optimizing treatment regimens. The key difference between hypertension specialist care and usual care was the systematic diagnosis and treatment of hypertension, particularly the identification and intervention of secondary hypertension. All specialists involved had extensive experience, and detailed descriptions of the center, specialists, and care procedures are provided in the Supplementary Material (Supplementary Methods and Supplementary Figure S1).

We identified patients based on their admission or discharge departments in the electronic medical records between 1 January 2015 and 31 December 2019, determined whether they had received medical care from hypertension specialists, and then divided them into the two groups. Hypertension specialists group: Patients with at least one hospitalization at the hypertension center within the aforementioned period were enrolled in the hypertension specialists group. The index date was defined as the date of initial admission to the hypertension center. Non-specialists group: Patients with no record of hospitalization at the hypertension center throughout the aforementioned period and who received usual care from non-specialists in the same clinical setting during the same period were enrolled into the non-specialists group. Physicians in the non-specialists group comprised those specializing in Cardiology, Nephrology, Endocrinology, Neurology, General Medicine, and other internal medicine subspecialties. The index date was defined as the date of initial admission to the non-specialists department. The follow-up period began on the index date and ended on 30 April 2023.

### 2.4 Outcomes

The primary outcome of this analysis was a composite of the first occurrence of MACE from the index date through the follow-up period, which included cardiovascular death, non-fatal stroke (ICD-10: I60-I63), non-fatal myocardial infarction (ICD-10:I21-I22), and coronary revascularization (22–24). The secondary outcomes included the individual components of the primary composite outcome. Coronary revascularization was defined as percutaneous coronary intervention with stent implantation and coronary artery bypass grafting (CABG), identified by comparing the surgical records with the medical records. Deaths were classified into cardiovascular and non-cardiovascular categories, and the date of cardiovascular death was obtained from provincial death records. Cardiovascular death was defined as being attributed to a cardiovascular etiology, including acute myocardial infarction, stroke, heart failure, malignant arrhythmia, sudden cardiac death, or other cardiovascular causes (pulmonary embolism and aortic dissection). Definitions of study outcomes are provided in the online Supplementary Material (Supplementary Methods). Data on MACE diagnosis during the follow-up were primarily obtained from UHDATA, national physical examination records, and the social medical insurance system of Urumqi. For participants who experienced these outcomes multiple times during the follow-up period, only the first occurrence was used for the analysis of MACE. Patients were observed from their index date until the occurrence of MACE or the last follow-up, which was censored as of 30 April 2023.

### 2.5 Statistical analysis

Continuous variables are presented as mean ± SD or median [interquartile range (IQR)], while categorical variables are presented as numbers (percentage). Statistical comparisons between groups used the independent Student's *t*-test or Mann–Whitney test for continuous data, and the Pearson's chi-square test for categorical data, as appropriate.

The cumulative incidence of cardiovascular outcomes was estimated via the Kaplan–Meier method and compared using the log-rank test. Cox proportional hazards regression models were used to estimate hazard ratio (HRs) along with 95% confidence intervals (CIs) for the cardiovascular outcomes. The proportional hazards assumption was checked by examining plots of the scaled Schoenfeld residuals against time for the covariates (GLOBAL *P* = 0.543). The adjusted model included sex, age, BMI, systolic BP, diastolic BP, marital status, occupation, duration of hypertension, antihypertensive therapy, current smoker, current drinker, TG, LDL-c, HDL-c, UA, and comorbidities at baseline. Meanwhile, win ratio analyses were conducted to assess the effect of medical care by hypertension specialists on the hierarchical composite outcome of cardiovascular death > non-fatal stroke > non-fatal myocardial infarction > coronary revascularization (the “>” symbol denotes the order of the win ratio hierarchy, which decreases from left to right). In addition, subgroup analyses were performed to further evaluate the association between care by hypertension specialists and outcomes based on sex, age, BMI, current smoking status, current drinking status, duration of hypertension, and type of hypertension. Furthermore, sensitivity analyses were performed to verify the robustness of results by excluding participants with MACE ≤1 year, IHD, CCH, CKD, or DM at baseline. A competing risk analysis using Fine–Gray modeling (25, 26) was also performed to estimate unadjusted and multivariable-adjusted subdistribution hazard ratios (sHRs) and 95% CI after sIPTW for MACE, accounting for non-cardiovascular death as a competing risk. E-values were calculated to assess the effect of unmeasured confounding variables on the results (27). Missing data for baseline covariables were handled using multiple imputations based on the method of chained equations. Five imputation datasets were created, with 0.14% of the covariables imputed (Supplementary Table S2). No outcomes were imputed. The propensity score method of stabilized inverse probability of treatment weighting (sIPTW) was used to ensure a similar distribution of baseline characteristics between the two groups (28). All variables in the multiple Cox regression model were checked for multicollinearity, which was defined as variance inflation factor >5 and tolerance <0.10 (Supplementary Table S5). Multiple imputation, Cox proportional hazards regression, inverse probability of treatment weighting, and win ratio analyses were performed using SPSS 26.0 and R (version 4.2.2) statistical software; all analyses were two-tailed, and *p* < 0.05 was considered statistically significant.

## 3 Results

### 3.1 Baseline characteristics of the cohort

**Figure 1:**
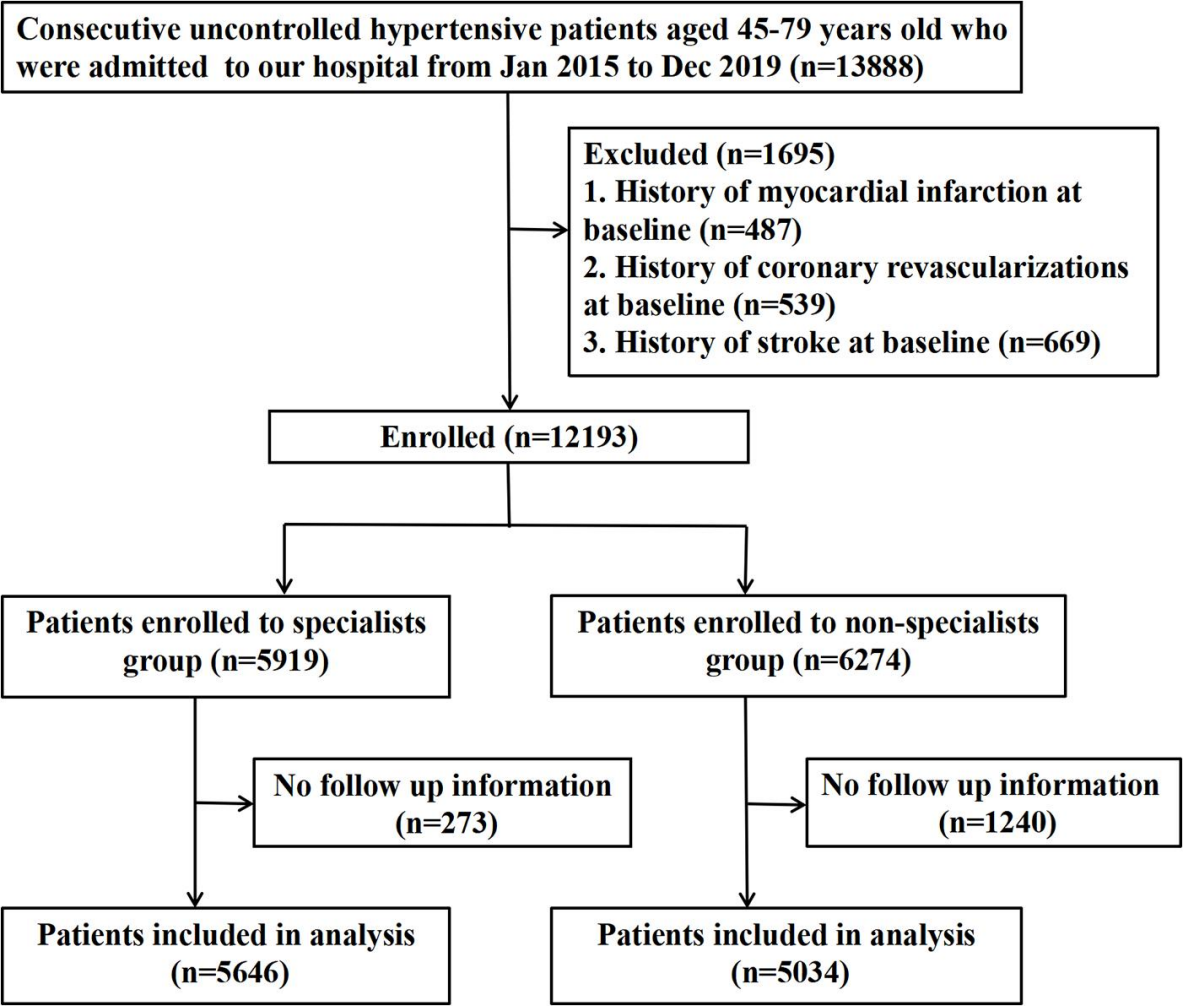
Flowchart of participant identification and inclusion criteria.

Between 1 January 2015 and 31 December 2019, a total of 10,680 (87.6%) participants completed follow-up and were enrolled in the analysis, including 5,646 (52.9%) participants in the specialists group and 5,034 (47.1%) in the non-specialists group (selection of the study population is shown in Figure 1). The mean age of the cohort was 59.4 years (SD 9.6), and 51.2% (5,472) of the participants were men (Table 1). The differences in the clinical characteristics between the two groups when admitted are shown in Table 1. Notably, before sIPTW, patients in the specialists group had significantly higher baseline BP (systolic BP, 152.2 ± 16.9 mmHg vs. 146.5 ± 16.8 mmHg, *p* < 0.001), TC (4.6 ± 1.0 mmol/L vs. 4.5 ± 1.1 mmol/L, *p* < 0.05), LDL-c (2.8 ± 0.8 mmol/L vs. 2.7 ± 0.9 mmol/L, *p* < 0.001), and UA (338.6 ± 88.1 mmol/L vs. 328.4 ± 92.0 mmol/L, *p* < 0.001) values compared to the non-specialists group. After sIPTW, there were no meaningful differences in the distribution of measured baseline clinical characteristics between the two groups, with all maximum absolute standardized differences <0.1 (Table 1). Other baseline characteristics between the two groups are provided in the Supplementary Material (Supplementary Tables S3, S4).

**Table 1:** Baseline characteristics of the study population.

	Before sIPTW					After sIPTW			
	Total cohort	Specialists group	Non-specialists group			Specialists group	Non-specialists group		
Characteristics	(*n* = 10,680)	(*n* = 5,646)	(*n* = 5,034)	*p*-Value*	SMD**	(*n* = 5,718.1)	(*n* = 5,042.7)	*p-*Value*	SMD**
Male, no. (%)	5,472 (51.2)	2,967 (52.6)	2,505 (49.8)	0.004	0.056	2,894.9 (50.7)	2,550.1 (50.5)	0.88	0.005
Age, mean (SD), years	59.4 (9.6)	56.8 (8.9)	62.4 (9.4)	<0.001	0.612	59.8 (9.8)	59.6 (9.3)	0.55	0.019
Married, no. (%)	10,302 (96.5)	5,460 (96.7)	4,842 (96.2)	0.16	0.028	5,537.6 (96.8)	4,874.5 (96.7)	0.66	0.010
Occupation, no. (%)									
Employed	4,226 (39.6)	2,697 (47.8)	1,534 (30.5)	<0.001	0.374	2,208.9 (38.6)	1,938.0 (38.4)	0.64	0.026
Retired	4,627 (43.3)	2,026 (35.9)	2,602 (51.7)			2,582.3 (45.2)	2,238.9 (44.4)		
Unemployed/homemaker	1,827 (17.1)	923 (16.3)	898 (17.8)			926.9 (16.2)	865.9 (17.2)		
Current smoking status, no. (%)	2,060 (19.3)	1,264 (22.4)	796 (15.8)	<0.001	0.168	1,068.4 (18.7)	944.0 (18.7)	0.97	0.001
Current drinking status, no. (%)	2,292 (21.5)	1,451 (25.7)	841 (16.7)	<0.001	0.221	1,189.9 (20.8)	1,081.1 (21.4)	0.60	0.015
Comorbidities, no. (%)									
Ischemic heart diseases	2,397 (22.4)	614 (10.9)	1,783 (35.4)	<0.001	0.608	1,403.6 (24.6)	1,150.2 (22.8)	0.21	0.041
Chronic cerebral hypoperfusion	5,496 (51.5)	3,698 (65.5)	1,798 (35.7)	<0.001	0.624	3,005.4 (52.7)	2,641.4 (52.4)	0.90	0.004
Chronic kidney diseases	505 (4.7)	217 (3.8)	288 (5.7)	<0.001	0.088	309.7 (5.4)	248.7 (4.9)	0.53	0.022
Diabetes	3,487 (32.6)	1,186 (21.0)	2,301 (45.7)	<0.001	0.543	1,979.5 (34.6)	1,633.4 (32.4)	0.12	0.047
Duration of hypertension, median (IQR), years	5.8 (2.0, 10.0)	5.0 (1.0, 10.0)	6.0 (2.0, 11.0)	<0.001	0.113	6.0 (2.0, 13.0)	6.0 (2.0, 10.0)	0.14	0.053
Antihypertensive therapy, no. (%)	6,742 (63.1)	3,922 (69.5)	2,820 (56.0)	<0.001	0.281	3,758.8 (65.8)	3,215.6 (63.7)	0.10	0.045
Clinical characteristics, mean (SD)									
Systolic BP (mmHg)	149.5 (17.1)	152.2 (16.9)	146.5 (16.8)	<0.001	0.339	149.5 (16.8)	150.0 (18.5)	0.44	0.030
Diastolic BP (mmHg)	87.7 (12.5)	89.8 (12.9)	85.3 (11.7)	<0.001	0.368	87.3 (13.3)	87.6 (12.6)	0.52	0.023
Fasting glucose (mmol/L)	5.8 (2.5)	5.4 (1.9)	6.4 (2.9)	<0.001	0.410	6.0 (3.1)	5.8 (2.4)	0.28	0.053
Triglyceride (mmol/L)	1.9 (1.5)	1.9 (1.5)	1.9 (1.5)	0.55	0.012	1.9 (1.5)	1.9 (1.4)	0.51	0.018
Total cholesterol (mmol/L)	4.6 (1.1)	4.6 (1.0)	4.5 (1.1)	0.037	0.040	4.6 (1.1)	4.5 (1.1)	0.58	0.018
LDL-cholesterol (mmol/L)	2.7 (0.9)	2.8 (0.8)	2.7 (0.9)	<0.001	0.082	2.7 (0.8)	2.7 (0.9)	0.92	0.003
HDL-cholesterol (mmol/L)	1.1 (0.3)	1.0 (0.3)	1.1 (0.3)	0.32	0.019	1.1 (0.3)	1.1 (0.3)	0.93	0.002
Uric acid (μmol/L)	333.7 (90.1)	338.6 (88.1)	328.4 (92.0)	<0.001	0.113	335.3 (87.9)	334.7 (94.1)	0.83	0.007
Body mass index (kg/m^2^)	26.9 (3.8)	26.8 (3.7)	27.0 (4.0)	0.020	0.045	27.0 (3.8)	26.9 (3.9)	0.63	0.015
eGFR (mL/min per 1·73 m^2^)	96.8 (12.2)	99.2 (10.4)	94.2 (13.4)	<0.001	0.418	96.2 (11.5)	96.6 (13.1)	0.35	0.029

Data are expressed as mean ± SD, median (IQR), and number (percentage) as appropriate. BP, blood pressure; LDL-cholesterol, low-density lipoprotein cholesterol; HDL cholesterol, high-density lipoprotein cholesterol; eGFR, estimated glomerular filtration rate; sIPTW, stabilized inverse probability of treatment weighting; SMD, standardized mean difference.

* *p*-Values for categorical data generated using the Pearson's chi-square test for two independent proportions and for numeric data using the Student's *t*-test or Mann–Whitney *U*-test for two independent groups.

** SMD <0.1 indicates balance between groups.

### 3.2 Association between medical care by hypertension specialists and the risk of MACE

**Figure 2:**
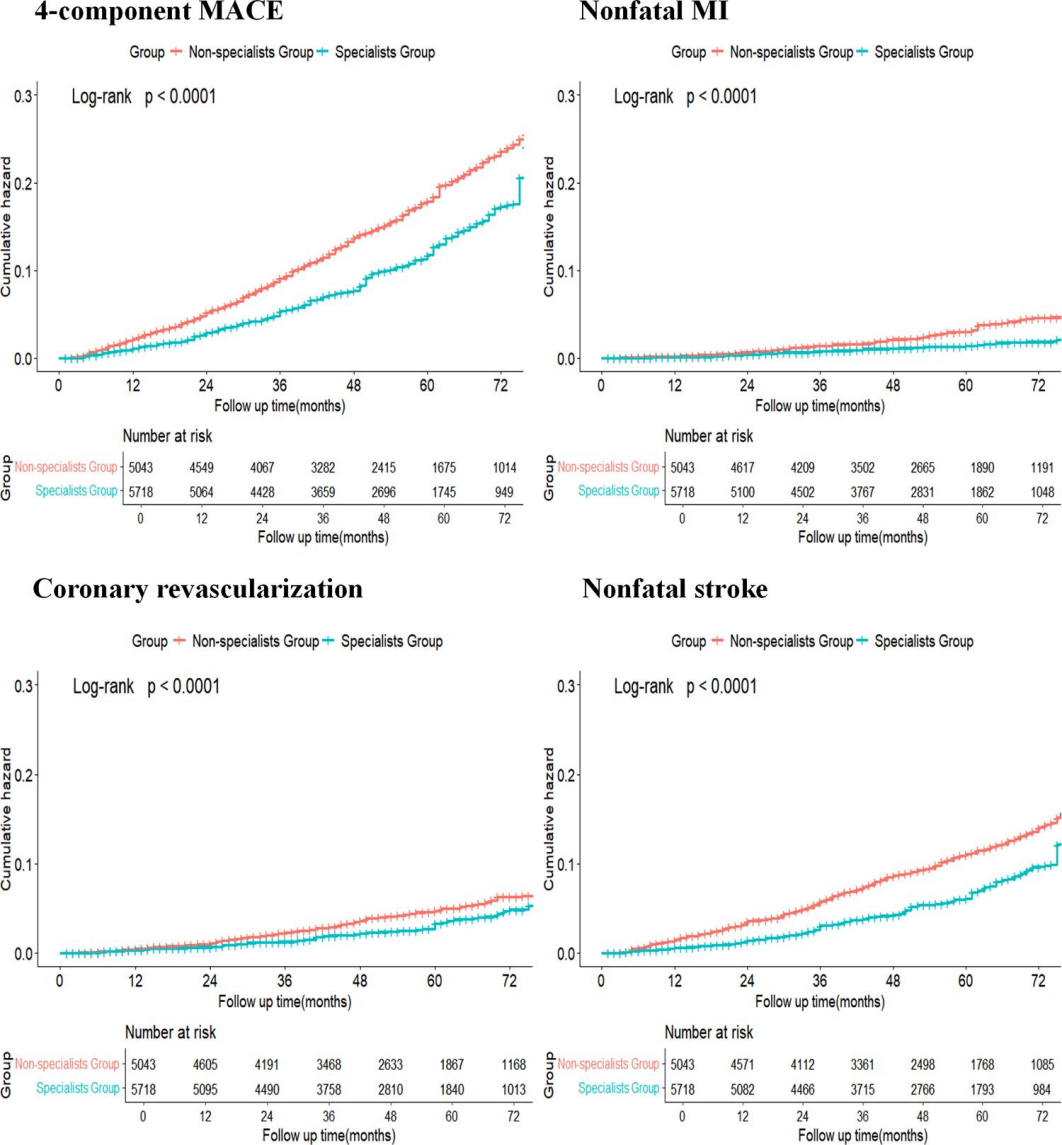
Kaplan–Meier curve comparing the specialists group and the non-specialists group after sIPTW. Blue line indicates cumulative hazard for the specialists group after sIPTW; orange line indicates cumulative hazard for the non-specialists group after sIPTW.

Table 2 shows the relationship between hypertension specialist medical care and four-component MACE. During a median follow-up of 4.0 years (interquartile range, 2.4–5.7 years), the primary outcome was confirmed in 439 participants (21.5 per 1,000 person-years) in the hypertension specialists group and 806 participants (39.7 per 1,000 person-years) in the non-specialists group. The difference between the two groups was statistically significant before and after sIPTW (log-rank test, *p* < 0.001, Figure 2). The secondary outcomes included 753 cases of non-fatal stroke, 216 cases of non-fatal myocardial infarction, 362 cases of coronary revascularization, and 118 cases of cardiovascular death. Similar significant differences were also observed for secondary outcomes, including non-fatal stroke (12.2 vs. 24.0 per 1,000 person-years), non-fatal myocardial infarction (3.1 vs. 7.0 per 1,000 person-years), and coronary revascularization (6.4 vs. 10.7 per 1,000 person-years), except for cardiovascular death (log-rank test, *p* < 0.001, Figure 2). The risk of MACE was significantly lower in the hypertension specialists group (HR after sIPTW = 0.73, 95% CI 0.61–0.87, *p* < 0.001), and the association was stable and became stronger after the multiple Cox regression analysis (adjusted HR = 0.67, 95% CI 0.57–0.79, *p* < 0.001). A similar tendency was observed for secondary outcomes (all *p* < 0.05), except for cardiovascular death.

**Table 2:** Unadjusted and adjusted hazard ratio of major adverse cardiovascular events between specialists group and non-specialists group after sIPTW.

	Specialists group (*n* = 5,646)		Non-specialists group (*n* = 5,034)					
Outcomes^a^	Cases with event, *n* (%)	Incidence rate (cases/1,000 person years)	Cases with event, *n* (%)	Incidence rate (cases/1,000 person years)	Unadjusted HR (95% CI)	*p*-Values	Adjusted HR^b^(95% CI)	*p*-Values
Primary outcome								
Four-component MACE^c^	439 (7.8)	21.5	806 (16.0)	39.7	0.73 (0.61–0.87)	<0.001	0.67 (0.57–0.79)	<0.001
Secondary outcomes								
Non-fatal stroke	253 (4.5)	12.2	500 (9.9)	24.0	0.69 (0.53–0.89)	0.001	0.62 (0.49–0.79)	<0.001
Non-fatal myocardial infarction	65 (1.2)	3.1	151 (3.0)	7.0	0.47 (0.33–0.68)	<0.001	0.48 (0.33–0.69)	<0.001
Coronary revascularization	133 (2.4)	6.4	229 (4.5)	10.7	0.73 (0.55–0.96)	0.027	0.71 (0.55–0.93)	0.012
Cardiovascular death	43 (0.8)	2.0	75 (1.5)	3.4	1.47 (0.83–2.60)	0.19	1.31 (0.74–2.32)	0.35

MACE, major adverse cardiovascular events; sIPTW, stabilized inverse probability of treatment weighting; CI, confidence interval; HR, hazard ratio.

a The number (%) of people with at least one event.

b Adjusted for sex, age, baseline body-mass index, systolic BP, diastolic BP, duration of hypertension, married, occupation, antihypertensive therapy, current smoker, current drinker, triglyceride, low-density lipoprotein cholesterol, high-density lipoprotein cholesterol, uric acid, and baseline comorbidities.

c Four-component MACE including non-fatal stroke, non-fatal myocardial infarction, coronary revascularization, and cardiovascular death.

Supplementary Figure S2 demonstrates the main analysis of the win ratio. All patients in the specialists group were paired with all patients in the non-specialists group, resulting in 5,646 × 5,034 = 28,421,964 patient pairs. The specialists group had a win proportion of 8.89%, whereas the non-specialists group had a win proportion of 4.65%. The specialists group had an adjusted win ratio of 1.91 over the non-specialists group (95% CI 1.69–2.16, *p* < 0.001) after sIPTW (Supplementary Figure S2).

### 3.3 Subgroup analyses between medical care by hypertension specialists and the risk of MACE

**Figure 3:**
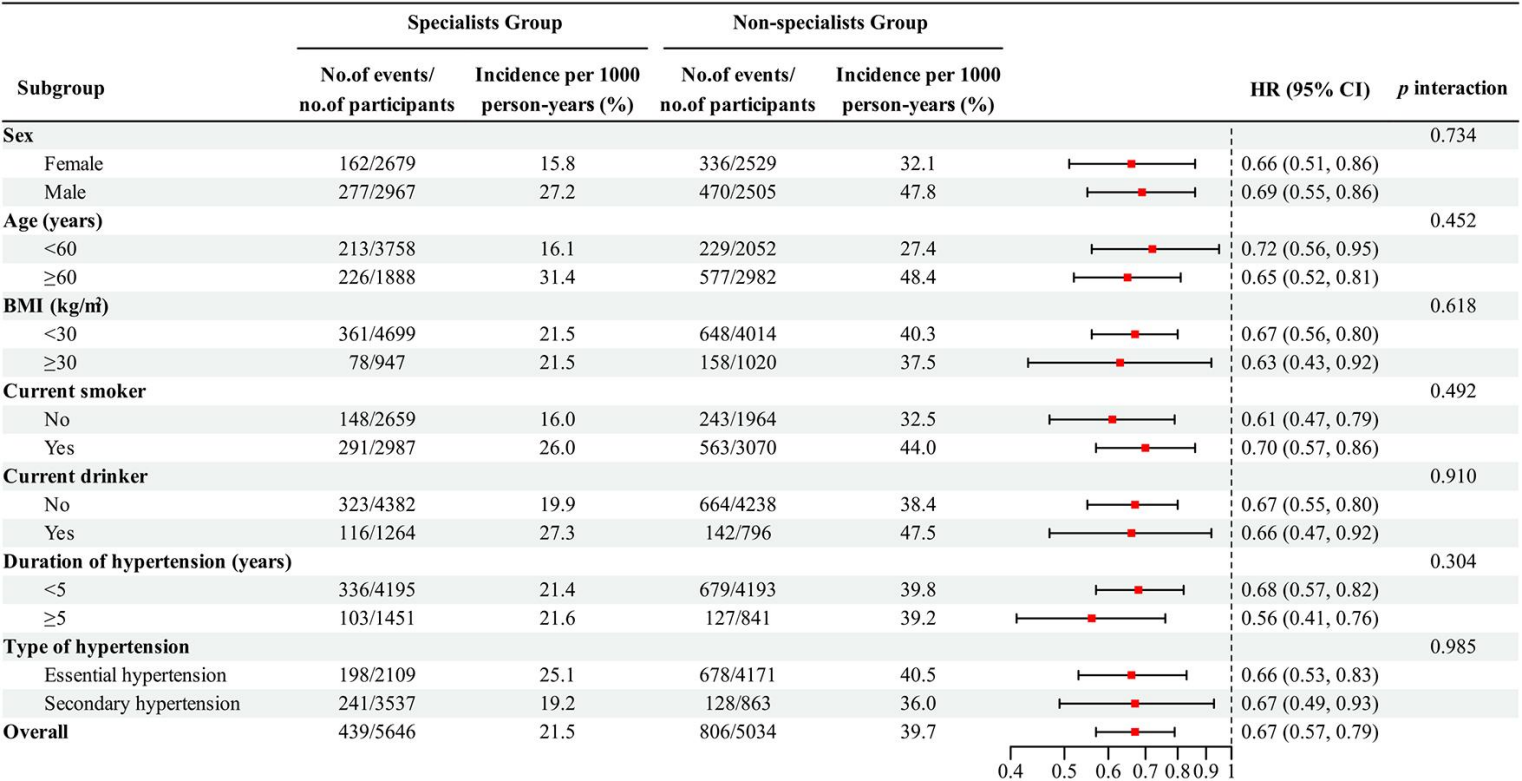
Subgroup analysis of major adverse cardiovascular events in patients with uncontrolled hypertension after sIPTW.

Figure 3 shows subgroup analyses assessing the effect of potential confounders, which might affect the relationship between care by hypertension specialists and the risk of MACE. The findings mentioned previously are consistent across subgroups categorized by age, sex, BMI, duration of hypertension, current smoking status, current drinking status, and type of hypertension at baseline, with no significant interaction effect noted (*p* for all interactions >0.05, Figure 3). Similar results were observed for secondary outcomes (Supplementary Figures S4–S6).

### 3.4 Sensitivity analyses between medical care by hypertension specialists and the risk of MACE

In all sensitivity analyses, the association between medical care by hypertension specialists and the risk of MACE remained robust and consistent (Supplementary Tables S6, S7). In the competing risk analysis accounting for non-cardiovascular death as a competing event, medical care by hypertension specialists remained significantly associated with a reduced risk of MACE (adjusted sHR = 0.63, 95% CI: 0.55–0.72, *p* < 0.001) (Supplementary Table S8). This sHR indicated that non-cardiovascular death did not substantially alter the association between care by hypertension specialists and MACE risk.

The *E*-value for unmeasured confounding in the multiple Cox regression examining the association between care by hypertension specialists and MACE was 2.35 for the point estimate and 1.85 for the lower bound of the 95% CI (Supplementary Table S9). The *E*-value indicates that our primary finding is robust to moderate unmeasured confounding and unlikely to be driven by residual bias from uncollected variables.

## 4 Discussion

In this real-world retrospective cohort study, we found that patients receiving care from hypertension specialists had a 33% lower risk of MACE compared with those receiving usual care from the non-specialists. In addition, care provided by hypertension specialists was associated with a decreased incidence of the secondary outcomes, including a 38% reduction in non-fatal stroke, a 52% reduction in non-fatal myocardial infarction, and a 29% reduction in coronary revascularization. Subgroup analyses further confirmed that both patients with essential and secondary hypertension significantly benefited from care provided by hypertension specialists. These findings validate the clinical value of hypertension specialist care in high-risk populations and extend the evidence from previous studies on hypertension management strategies.

Existing multilevel strategies led by non-physicians (e.g., village doctors, nurses) have been shown to effectively improve BP control and cardiovascular outcomes in general hypertensive populations within primary care settings (29–31). Our study extends this evidence by focusing on patients with uncontrolled hypertension, a high-risk subgroup for whom routine strategies may be insufficient due to complex clinical challenges like drug resistance or undetected secondary causes. The observed cardiovascular risk reduction underscores that hypertension specialist care serves as an advanced, multicomponent intervention tailored to the unmet needs of this challenging population. Prior studies established that referral to hypertension specialists improves BP control, supporting the value of specialist engagement (15, 17). Our study builds upon this by focusing on MACE, demonstrating that such care also translates into tangible long-term cardiovascular benefits.

Three interrelated mechanisms may explain the observed benefits: The first benefit is the strict adherence to guideline-directed evidence-based practice. Specialists demonstrate better adherence to evidence-based practice (32, 33) and excel at optimizing and refining antihypertensive regimens, which positively affects the patient's adherence to treatment (34). The second benefit is timely identification and management of the underlying causes of secondary hypertension. Secondary hypertension is associated with an increased risk of cardiovascular disease and more severe target organ damage, with certain forms being independent of blood pressure control (35, 36). Patients with suspected secondary hypertension are more readily identified and diagnosed in hypertension specialist settings (37, 38). Despite a higher secondary hypertension diagnosis rate (62.65%) in the specialists group, the associated risk of MACE was effectively mitigated, supporting the notion that identifying the underlying cause of secondary hypertension may lead to successful interventions targeting the potential etiology and reducing cardiovascular morbidity (39). Since certain forms of secondary hypertension are often asymptomatic, diagnostic delay or misdiagnosis in routine clinical practice is particularly common in non-specialist settings, consequently leading to preventable end-organ injury and poorer clinical outcomes (40, 41). The third benefit is comprehensive management of clustered risk factors. Hypertension specialists achieve both superior BP control (15, 17) and address other modifiable cardiovascular risk factors (18). Despite higher baseline risk profiles (TG, TC, LDL-c, and UA), patients under specialist care achieved a 52% lower risk of myocardial infarction, indicating care benefits beyond mere BP reduction.

Our findings support a stratified management approach: Routine hypertension cases can be effectively managed in primary care settings, while patients with uncontrolled BP or suspected complexity should be referred to specialists. This strategy addresses knowledge gaps among frontline providers and ensures that high-risk patients receive optimized care. From a public health perspective, given the global burden of uncontrolled hypertension, incorporating the identification and intervention of secondary hypertension into hypertension care through specialists could meaningfully diminish the population-level burden of cardiovascular disease.

The study has several key strengths that reinforce its findings and implications. First, it used a large cohort sample size and longer follow-up time, which increased the generalizability and statistical power of the results. Second, the research findings demonstrated robustness and consistency across a comprehensive range of sensitivity and subgroup analyses, thereby enhancing confidence in their reliability. Third, the study's patient-centered approach, focused on individuals with uncontrolled hypertension, uniquely identified the specific benefits of specialist medical care in reducing the risk of MACE and demonstrated the substantial clinical value of such specialist care within a high-risk CVD population. Taken together, these strengths highlight the significant contribution of the study to clinical practice and health policy for individuals with hypertension.

This study has several limitations. First, the single-center, retrospective design of this study may restrict causal inference and the generalizability of our findings. Future multicenter studies covering both tertiary and community settings could further validate our conclusions. Second, while the sIPTW adjustment successfully balanced baseline confounding between the two groups, unmeasured or residual confounding may still exist due to the lack of data on socioeconomic status (e.g., income level, educational attainment), health behaviors, and medication adherence, and these factors might affect the study findings. For instance, patients with higher socioeconomic status may be more likely to seek specialist care, which positively affects the patient's adherence to treatment; these factors could also contribute to improved cardiovascular outcomes. *E*-value was calculated to assess the robustness of our findings to unmeasured confounding, and 2.35 was considered modestly robust, suggesting that the effect of unmeasured confounders is unlikely to alter the findings. Third, exposure status was only assessed at baseline. Furthermore, due to the limitations of the UHDATA Database, we were unable to evaluate whether patients sought care at the hypertension departments of other medical institutions after the index date, which may have led to an underestimation of the effect observed in our study.

## 5 Conclusion

In conclusion, this study demonstrated a significant association between medical care provided by hypertension specialists and a reduced risk of MACE in patients with uncontrolled hypertension. Our findings suggest that hypertension specialist care might play an essential role in improving cardiovascular outcomes among this high CVD risk population.

## Data Availability

The original contributions presented in the study are included in the article/Supplementary Material, further inquiries can be directed to the corresponding author.

## The Members of Hypertension Specialists Medical Care Study Research Group

De-lian Zhang, Qin Luo, Meng-hui Wang, Jun-li Hu, Guo-liang Wang, Ying-chun Wang, Nuerguli Maimaiti, Ke-ming Zhou, Lei Wang, Xiao-guang Yao, Wen Jiang, Le Sun.
