# Supplementary material for: Association between care by hypertension specialists and major adverse cardiovascular events in patients with uncontrolled hypertension
Source: Front Cardiovasc Med. 2026 Feb 9;13:1697092. doi: 10.3389/fcvm.2026.1697092 (PMC12926387; doi:10.3389/fcvm.2026.1697092)
Supplement: Supplementary file 1 [file Datasheet1.docx]

***Supplementary Material****

**Contents Page**

**[Supplementary Material* 1](#_Toc5968)**

**[Supplementary Methods 3](#_Toc27966)**

[The detailed information on UHDATA 3](#_Toc15347)

The detailed description of the Hypertension Center in People’s Hospital of Xinjiang

Uygur Autonomous Region 3

The detailed description of the procedures for care by hypertension specialists......... 4

Definitions of MACE outcomes 5

Statistical Analysis of win ratio 6

**[Supplementary Figures 8](#_Toc15)**

[Figure S1. The procedures for care by hypertension specialists 8](#_Toc10448)

Figure S2. Adjusted win ratio analysis of MACE in patients with uncontrolled hypert

-ension after sIPTW 9

[Figure S3. Subgroup analysis of nonfatal stroke in patients with uncontrolled hyperte](#_Toc13134)

[-nsion after sIPTW](#_Toc13134) 10

[Figure S4. Subgroup analysis of nonfatal myocardial infarction in patients with uncon](#_Toc24664)

[-trolled hypertension after sIPTW 1](#_Toc24664)1

[Figure S5. Subgroup analysis of coronary revascularization in patients with uncontrol](#_Toc28599)

[-led hypertension after sIPTW](#_Toc28599) 12

**[Supplementary Tables 1](#_Toc18713)3**

[Table S1. Baseline Characteristic of the included and no follow-up information partic](#_Toc11488)

[ipants.. 1](#_Toc11488)3

[Table S2. The description of missing data 14](#_Toc18932)

[Table S3. Detection of secondary causes and coexisting diseases in patients with unc -ontrolled hypertension](#_Toc8031) ..15

[Table S4. Medication before and after exposure at baseline 16](#_Toc2516)

[Table S5. Collinearity diagnostics steps 1](#_Toc29186)7

[Table S6. Sensitivity analysis of the relationship between care by hypertension speci](#_Toc19392)

[-alists and MACE after sIPTW 1](#_Toc19392)8

[Table S7. The relationship between care by hypertension specialists and MACE by ty](#_Toc4692)

[-pe of hypertension after sIPTW 1](#_Toc4692)9

[Table S8. Subhazards ratio and 95% CI for MACE using a Fine-Gray Proportional S](#_Toc11488)

[-ubhazards Model after sIPTW](#_Toc11488) 20

[Table S9. E-values for unmeasured confounding between medical care by hypertensi](#_Toc11488)

[-on specialists and MACE](#_Toc11488) 21

*****This supplementary material was provided by the authors to give readers further details on their article. The material was reviewed but not copyedited.

# Supplement**ary** Methods

## **The detailed information on UHDATA**

The Hypertension Database in Urumqi (UHDATA), was a database for hypertension created by the Key Laboratory for Hypertension Clinical Research and the National Health Committee of China, located at the Xinjiang Uygur Autonomous Region People’s Hospital. This database includes information on patients socio-demographic information, outpatient and inpatient visits, medical history, medication, laboratory test results, clinical conditions, cost information, etc., and can retrieve medical records information that meets the inclusion criteria and the exclusion criteria based on predefined fields. The database contains all patients diagnosed with hypertension at the above hospital since January 2004.

## **The detailed description of the Hypertension Center in People’s Hospital of Xinjiang Uygur Autonomous Region**

Since 1997, Hypertension Center of the People’s Hospital of Xinjiang Uygur Autonomous has gradually established a platform for secondary hypertension screening, diagnosis and treatment. Currently, Hypertension Center has 5 outpatient clinics, including General Outpatient, Specialized Service and Expert Outpatient Service, and has 180 beds distributed in 4 wards. On the basis of the large-scale comprehensive medical examination platform, the Hypertension Center has set up the hypertension-oriented physical examination unit(including Dynamic Blood Pressure Chamber, Fundus Photography Room, the Arterial Elastic Testing Room, OSA Monitoring Room and Vascular Intervention Room) and the department of biochemical inspection (including endocrine hormone test, vasoactive substances test, cardiovascular risk factor evaluation and genetic test. This Hypertension Center boasts a team of highly qualified specialists in hypertension, comprising 82 medical staff (30 physicians and 52 nurses), of whom 16 hold the position of associate chief physician or higher. Physicians providing care at the hypertension center, whose primary specialty is Cardiology, have completed a subspecialty training pathway (Internal medicine → Cardiology → Hypertension) within their ongoing clinical practice. They possess a master's degree or higher, hold a practicing physician qualification certification issued by the National Health Commission of the People's Republic of China (NHCC), and have more than five years of clinical experience in practice, qualifying them to manage patients independently. Specifically, these physicians specialize in the diagnosis and management of hypertension and associated disorders, including essential hypertension, secondary hypertension, resistant hypertension, hypertension and its cardio-cerebrovascular complications, as well as hypertension accompanied by comorbidities such as diabetes mellitus or chronic kidney disease. Furthermore, hypertension specialists diligently focus on screening and diagnosing for secondary hypertension within their clinical practice, integrate etiological interventions into their management strategies, and optimize treatment regimens in addition to usual care.

**The detailed description of the procedures for care by hypertension specialists**

All patients underwent a standardized assessment of their BP level (including office BP level, home BP level, and 24-hours ambulatory BP level), overall cardiovascular disease (CVD) risks, and hypertensive complications. Subsequently, hypertension specialists with over five years of clinical experience assess whether there are screening suspicion for secondary hypertension based on symptoms, signs, and routine examination findings, thereby initiating the screening and diagnostic process for secondary hypertension. Ultimately, ascertain the exact classification of hypertension (essential or secondary) and provide personalized management strategies customized to each patient's type of hypertension, cardiovascular risk factors, and hypertensive complications, in accordance with established guidelines. Advice is provided regarding diet, exercise, salt intake, alcohol moderation, and weight management to optimize their lifestyle. Follow-up visit plans were also provided for patients with essential hypertension and secondary hypertension.

## **Definitions of MACE outcomes**

1. Myocardial infarction is defined as a transient increase in the specific laboratory markers of myocardial necrosis (CK-MB or Troponin T), accompanied by ischemic symptoms and/or typical electrocardiogram signs (pathological Q-wave or ST-segment elevation or depression), the control medical records and ICD diagnostic codes include acute myocardial infarction (ICD-10: I21-I22). All cases of myocardial infarction were confirmed through laboratory examination and electrocardiogram.

2. Coronary revascularization includes percutaneous coronary intervention (PCI) with stent implantation and CABG with coronary artery bypass surgery, which were obtained by comparing the surgical records with the medical records.

3. The first occurrence of stroke, including symptomatic ischemic stroke and hemorrhagic stroke, was defined according to the World Health Organization Multinational Monitoring of Trends and Determinants in Cardiovascular Disease criteria: rapidly developing signs of focal (or global) disturbance of cerebral function lasting >24 h (unless interrupted by surgery or death) with no apparent non-vascular cause. The definition included patients presenting with clinical signs and symptoms suggestive of complete stroke, including ischemic stroke and hemorrhagic stroke, the control medical records and ICD diagnostic codes were subarachnoid hemorrhage (ICD-10: I60), intracerebral hemorrhage (ICD-10: I61), and ischemic stroke (ICD-10:I63), of which ischemic stroke with hemorrhage transformation was not cerebral hemorrhage. All stroke cases were diagnosed by computed tomography, and magnetic resonance imaging (including diffusion image).

4. Cardiovascular death was defined as being attributed to a cardiovascular etiology, including acute myocardial infarction, stroke, heart failure, malignant arrhythmia, sudden cardiac death, or other cardiovascular causes(pulmonary embolism and aortic dissection).

If participants experienced these outcomes more than once during the follow-up period, only the first outcome was used for the analysis of MACE. Each patient was observed from their index date to the date of incidence of MACE, or last follow-up censored until April 30, 2023.

## **Statistical Analysis of win ratio**

The win ratio is defined by the number of winners in the specialists group divided by the number of winners in the non-specialists group. Winners are determined as follows: the event hierarchy from most to least important was cardiovascular death, nonfatal stroke, nonfatal myocardial infarction and coronary revascularization. The CI and P values for win ratio analyses were calculated with R wins Package. This method uses multiple resamples (with replacement) from the study population and computes the win ratio statistic on each of these resamples.

# Supplemental Tables

Figure S1. The procedures for care by hypertension specialists

Hypertension specialists with ≥5 years of clinical experience assessed patients for clinical suspicion of secondary hypertension based on clinical symptoms, physical signs, and primary evaluation results

Management

*etiology-targeted treatment

*Individualized regimens optimization

*Lifestyle modification

*Follow-up protocol

Management

*Regimens optimization

*Lifestyle modifications:

Diet

Sleep

Exercise

Salt intake

Smoking/Alcohol

Weight management

Personality/psychology

*Follow-up protocol

Essential hypertension

Secondary hypertension

1. OSA
2. Renal parenchymal hypertension/renal vascular hypertension;
3. Endocrine hypertension: Primary aldosteronism; Pheochromocytoma; Cortisol hypertrophy (Cushing syndrome); thyroid disease, etc.
4. Psychological disorders;
5. Drug-induced hypertension;
6. Others

Classification Diagnosis of Hypertension

Further evaluation:

PSG, Angiogram/CTA, Renal ECT, biopsy, hormonal assessments, adrenal venous blood sampling to determine hormonal levels, assessments of psychological, etc.

Take a detailed history, physical examination, results of laboratory, assistant examination, etc. for primary evaluation :

1. 24h-ambulatory BP assessment
2. Cardiovascular risk assessment
3. Hypertensive complications assessment

BP initial assessment：office BP level、home BP level

Antihypertensive treatment regimens and medication adherence assessment

Evaluate patients with uncontrolled hypertension

Figure S2. Adjusted win ratio analysis of MACE in patients with uncontrolled hypertension after sIPTW

Specialists Group

N=5646

Patient Pairs

28421964

Non-specialists Group

N=5034

N=6859

0.79%

91.99%

1.28%

98.72%

98.09%

5.36%

2.65%

0.49%

0.63%

WINS

WINS

1.46%

Total wins：8.89%

97.66%

0.88%

Total wins：4.65%

TIES

Four-component MACE

1.Cardiovascular death

2.Nonfatal stroke

3.Nonfatal myocardial infarction

4.Coronary revascularization

sIPTW unmatched win ratio (95%CI) = 1.91 (1.69-2.16), *p* value < 0.0001

[Figure S3. Subgroup analysis of nonfatal stroke in patients with uncontrolled hypertension after sIPTW](#_Toc13134)


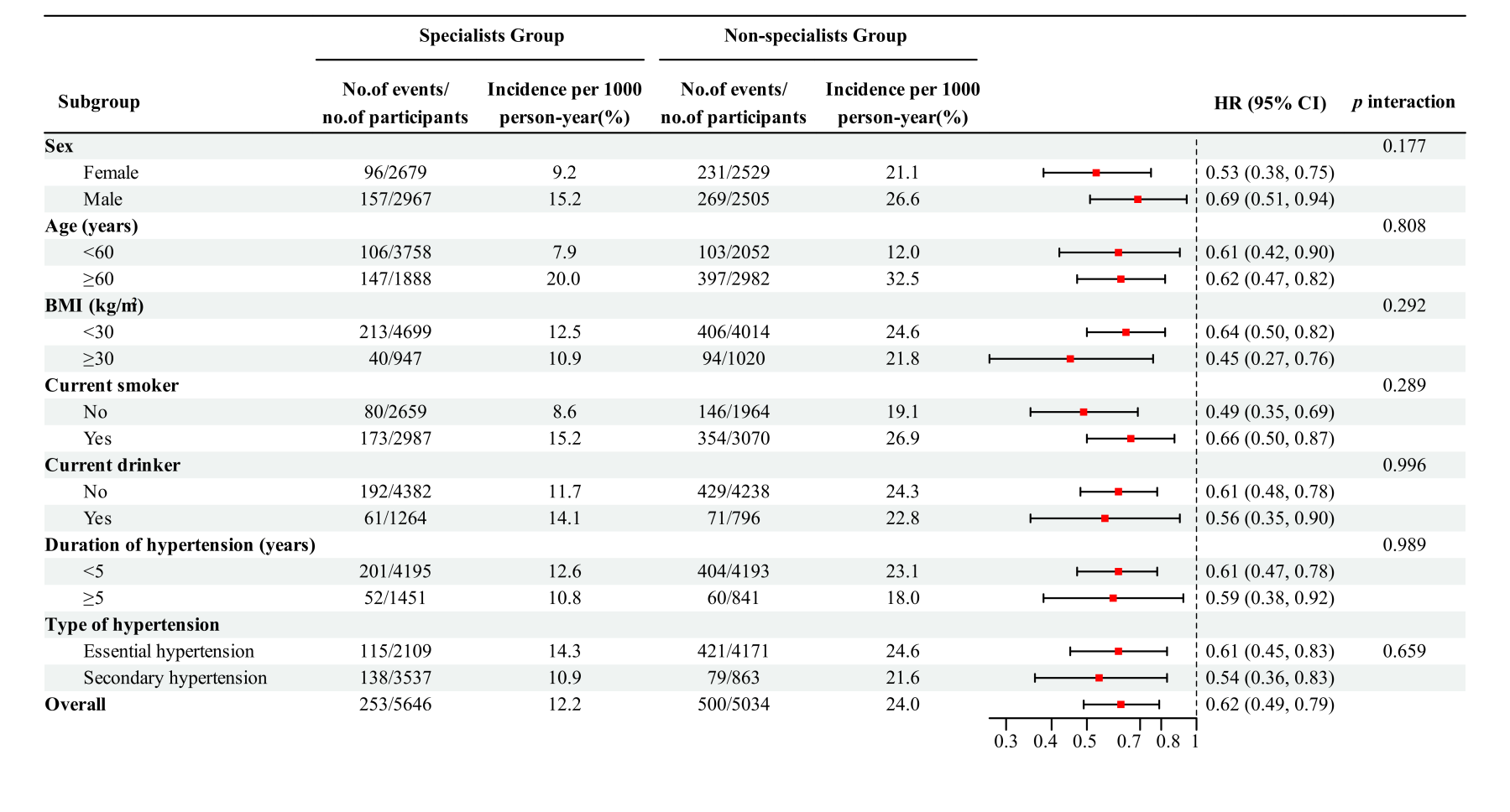


[Figure S4. Subgroup analysis of nonfatal myocardial infarction in patients with uncontrolled hypertension after sIPTW](#_Toc24664)

**
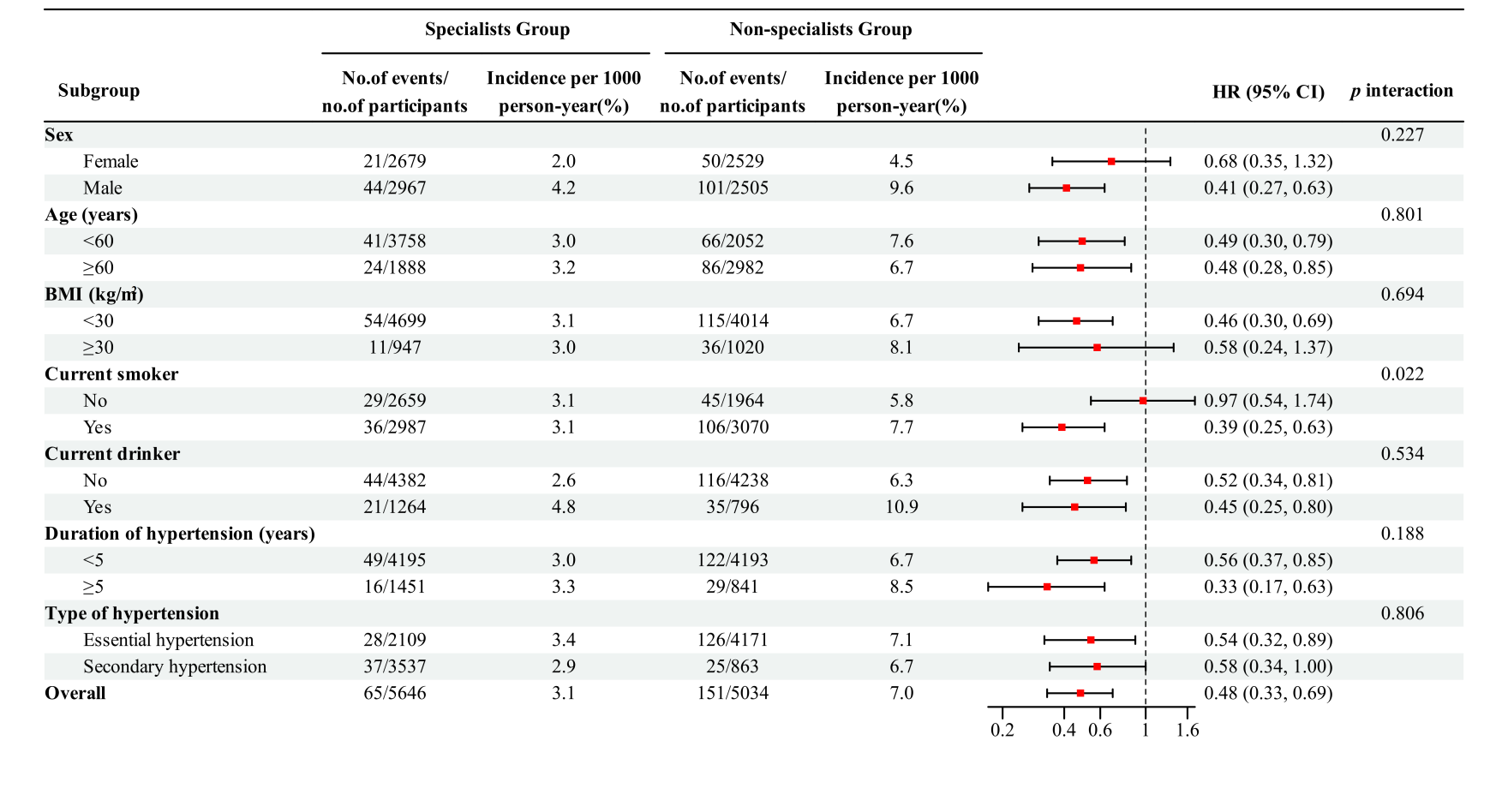
**

**[Figure S5. Subgroup analysis of coronary revascularization in patients with uncontrolled hypertension after sIPTW](#_Toc28599)**

**
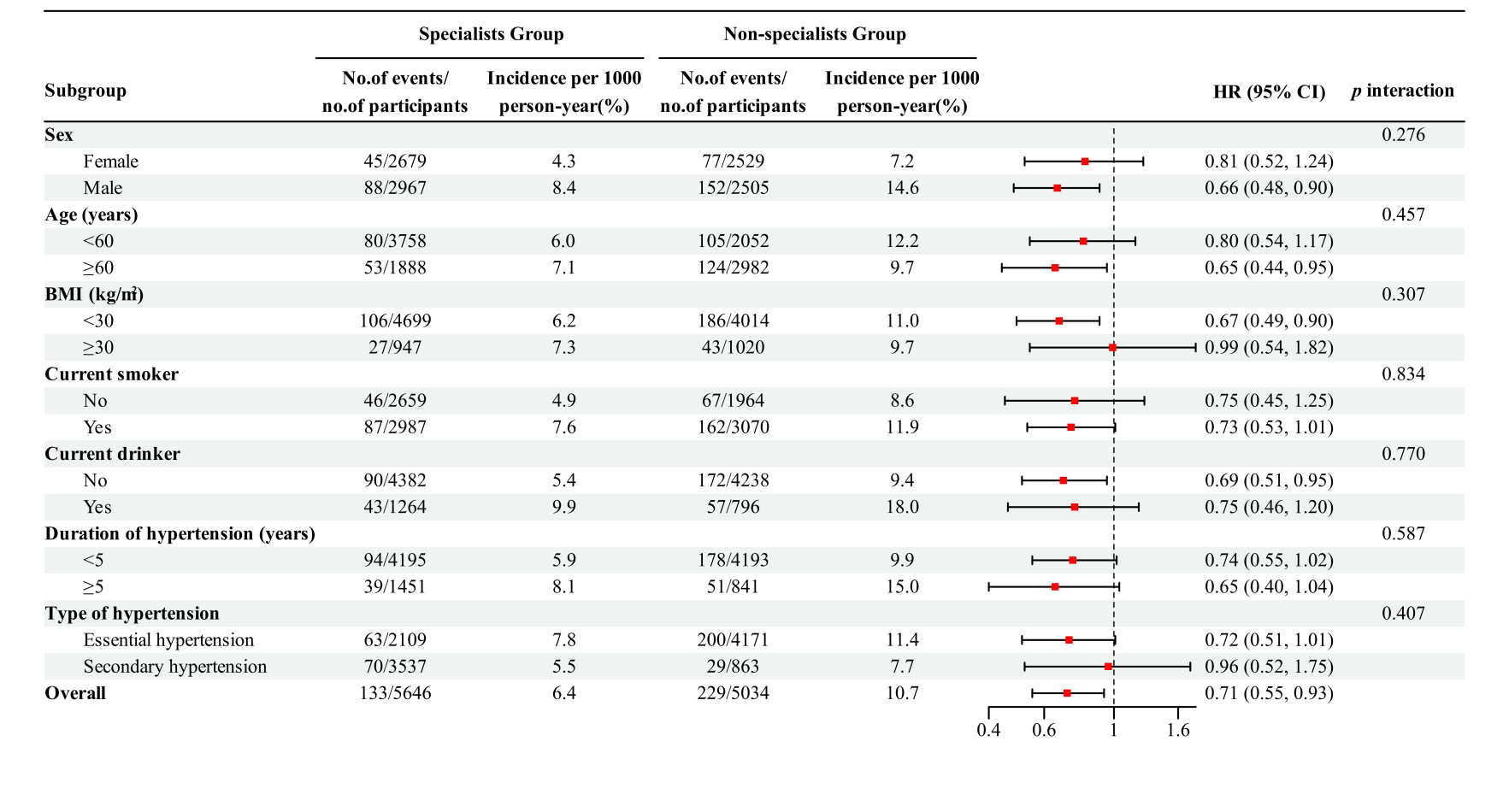
**

# Supplemental Tables

| Table S1. Baseline Characteristic of the included and no follow-up information participants | | | | |  |
| --- | --- | --- | --- | --- | --- |
|  | Overall | No follow-up information | Included |  | |
| Characteristics | (n = 12193) | (n = 1513) | (n = 10680) | *p* value ^a^ | |
| Male, n (%) | 6294(51.6) | 822(54.3) | 5472 (51.2) | <0.001 | |
| Mean age (SD), years | 59.7(9.7) | 61.8(10.0) | 59.4(9.6) | 0.026 | |
| Married, n (%) | 11739(96.3) | 1441(95.2) | 10298 (96.5) | 0.023 | |
| Occupation, n (%) |  |  |  | <0.001 | |
| Employed | 4583(37.7) | 382(25.2) | 4201 (39.5) |  | |
| Retired | 5103(42.0) | 491(32.5) | 4612 (43.4) |  | |
| Unemployed/Homemaker | 2456(20.2) | 640(42.3) | 1816 (17.1) |  | |
| Current smoker, n (%) | 2368(19.4) | 313(20.7) | 2055 (19.3) | 0.20 | |
| Current drinker, n (%) | 2589(21.3) | 304(20.1) | 2285 (21.4) | 0.25 | |
| Comorbidities, n (%) |  |  |  |  | |
| Ischemic heart diseases | 2873(23.6) | 476(31.5) | 2397 (22.4) | <0.001 | |
| Chronic cerebral hypoperfusion | 6201(50.9) | 705(46.6) | 5496 (51.5) | <0.001 | |
| Chronic kidney diseases | 567(4.7) | 62(4.1) | 505 (4.7) | 0.31 | |
| Diabetes | 3997(32.8) | 510(33.7) | 3487 (32.6) | 0.43 | |
| Median duration of hypertension, (IQR), years | 5.0(2.0, 10.0) | 5.0(0.7, 10.0) | 5.8(2.0, 10.0) | <0.001 | |
| Antihypertensive therapy, n (%) | 7976(65.4) | 1234 (81.6) | 6742 (63.1) | <0.001 | |
| Clinical characteristics |  |  |  |  | |
| Mean systolic BP(SD), mm Hg | 149.5(17.1) | 149.1(17.3) | 149.5(17.1) | 0.41 | |
| Mean diastolic BP(SD), mm Hg | 87.6(12.5) | 87.2(12.6) | 87.7(12.5) | 0.15 | |
| Mean fasting glucose (SD), mmol/L | 5.9(2.5) | 6.0(2.6) | 5.8(2.5) | 0.09 | |
| Mean triglyceride(SD), mmol/L | 1.9(1.5) | 1.9(1.5) | 1.9(1.5) | 0.93 | |
| Mean total cholesterol(SD), mmol/L | 4.5(1.1) | 4.4(1.1) | 4.5(1.1) | <0.001 | |
| Mean LDL-cholesterol(SD), mmol/L | 2.7(0.9) | 2.6(0.9) | 2.7(0.9) | <0.001 | |
| Mean HDL-cholesterol(SD), mmol/L | 1.1(0.3) | 1.1(0.3) | 1.1(0.3) | 0.06 | |
| Mean uric acid (SD), μmol/L) | 333.7(9.4) | 333.1(92.6) | 333.8(90.1) | 0.80 | |
| Mean body mass index (SD), kg/m^2^ | 26.8(3.8) | 26.6(3.9) | 26.9(3.8) | 0.02 | |
| Mean eGFR(SD), mL/min per 1∙73 m^2^ | 96.7(12.2) | 95.6(12.2) | 96.8(12.2) | <0.001 | |
| *BP* blood pressure; *LDL-cholesterol* low-density lipoprotein cholesterol; *HDL-cholesterol* high-density lipoprotein cholesterole; *GFR* estimated glomerular filtration rate; *sIPTW* stabilized inverse probability of treatment weighting; *SMD* standardized mean difference.  ^a^ *p* values for categorical data generated using the Pearson’s chi-square test for 2 independent proportions and for numeric data using the Student's t-test or Mann-Whitney U test for 2 independent groups. | | | | | |

| Table S2. The description of missing data | | |
| --- | --- | --- |
| Characteristics | Non-Missing | Missing |
| Male | 10680 | 0 |
| Age | 10680 | 0 |
| Married | 10676 | 4 |
| Occupation | 10629 | 51 |
| Current smoker | 10667 | 13 |
| Current drinker | 10667 | 13 |
| Comorbidities |  |  |
| Ischemic heart disease | 10680 | 0 |
| Chronic cerebral hypoperfusion | 10680 | 0 |
| Chronic kidney diseases | 10680 | 0 |
| Diabetes | 10680 | 0 |
| Duration of hypertension | 10680 | 0 |
| Antihypertensive therapy | 10680 | 0 |
| Clinical characteristics |  |  |
| Systolic BP | 10680 | 0 |
| Diastolic BP | 10680 | 0 |
| Fasting glucose | 10634 | 46 |
| Total cholesterol | 10638 | 42 |
| Triglyceride | 10638 | 42 |
| LDL-cholesterol | 10636 | 44 |
| HDL-cholesterol | 10636 | 44 |
| Uric acid | 10677 | 3 |
| Body mass index | 10635 | 45 |
| eGFR | 10677 | 3 |
| *BP* blood pressure; *eGFR* estimated glomerular filtration rate; *LDL-cholesterol* low-density lipoprotein cholesterol; *HDL-cholesterol* high-density lipoprotein cholesterol. | | |

| Table S3. Detection of secondary causes and coexisting diseases in patients with uncontrolled hypertension | | | | |
| --- | --- | --- | --- | --- |
| Diseases | Total | Specialists Group | Non-specialists Group | *p* value ^a^ |
| Secondary hypertension | 4400 (41.20) | 3537 (62.65) | 863 (17.14) | <0.001 |
| OSA | 1541 (14.43) | 1434 (25.40) | 107 (2.13) | <0.001 |
| Renal Hypertension | 73 (0.68) | 56 (0.99) | 17 (0.34) | <0.001 |
| Renal parenchymal hypertension | 51 (0.48) | 34 (0.60) | 17 (0.34) | 0.06 |
| Renovascular hypertension | 22 (0.21) | 22 (0.39) | 0 (0.00) | <0.001 |
| Endocrine hypertension | 1254 (11.74) | 1002 (17.75) | 252 (5.01) | <0.001 |
| Primary aldosteronism | 816 (7.64) | 790 (13.99) | 26 (0.52) | <0.001 |
| Cushing syndrome | 16 (0.15) | 14 (0.25) | 2 (0.04) | 0.012 |
| Pheochromocytoma | 2 (0.02) | 2 (0.04) | 0 (0.00) | 0.53 |
| Thyroid disease | 454 (4.25) | 227 (4.02) | 227 (4.51) | 0.23 |
| Hypothyroidism | 431 (4.04) | 208 (3.68) | 223 (4.43) | 0.06 |
| Hyperthyroidism | 26 (0.24) | 22 (0.39) | 4 (0.08) | 0.002 |
| Psychological disorders | 980 (9.18) | 646 (11.44) | 334 (6.63) | <0.001 |
| Others ^b^ | 2 (0.02) | 2 (0.04) | 0 (0.00) | 0.53 |
| Data are n (%).  OSA=obstructive sleep apnea.  ^a^ *p* values for categorical data generated using the Pearson’s chi-square test for 2 independent proportions.  ^b^ Others included coarctation of the aorta, takayasu arteritis, liddle syndrome. | | | | |

| Table S4. Medication before and after exposure at baseline | | | | | | | | | |
| --- | --- | --- | --- | --- | --- | --- | --- | --- | --- |
|  | **Before exposure , no. (%)** | | | |  | **After exposure , no. (%)** | | | |
|  |  | **Specialists** | **Non-specialists** |  |  |  | **Specialists** | **Non-specialists** |  |
| **Drugs** | **Total** | **Group** | **Group** | ***p*** **value** ^b^ |  | **Total** | **Group** | **Group** | ***p*** **value** ^b^ |
| **Antihypertensive agents** |  |  |  |  |  |  |  |  |  |
| **ACEI/ARB** | 451 (5.1) | 350 (7.6) | 101 (2.4) | <0.001 |  | 5829 (54.6) | 2527 (44.8) | 3302 (65.6) | <0.001 |
| **Beta blockers** | 1890 (21.4) | 1035 (22.3) | 855 (20.5) | 0.033 |  | 2484 (23.3) | 824 (14.6) | 1660 (33.0) | <0.001 |
| **Calcium channel blockers** | 5748 (65.2) | 3477 (75.0) | 2271 (54.3) | <0.001 |  | 7302 (68.4) | 3993 (70.7) | 3309 (65.7) | <0.001 |
| **Diuretics** | 1512 (17.2) | 1048 (22.6) | 464 (11.1) | <0.001 |  | 2581 (24.2) | 1833 (32.5) | 748 (14.9) | <0.001 |
| **MRA** | 291 (3.3) | 244 (5.3) | 47 (1.1) | <0.001 |  | 1424 (13.3) | 1187 (21.0) | 237 (4.7) | <0.001 |
| **Others** ^a^ | 345 (3.9) | 318 (6.9) | 27 (0.6) | <0.001 |  | 876 (8.2) | 790 (14.0) | 86 (1.7) | <0.001 |
| **Anti-platelet agents** | 1711 (19.4) | 707 (15.3) | 1004 (24.0) | <0.001 |  | 6576 (61.6) | 3275 (58.0) | 3301 (65.6) | <0.001 |
| **Lipid-lowering agents** | 1328 (15.1) | 614 (13.3) | 714 (17.1) | <0.001 |  | 8447 (79.1) | 4515 (80.0) | 3932 (78.1) | 0.020 |
| *ACEI/ARB* angiotensin-converting enzyme inhibitors/angiotensin receptor blockers; *MRA* Mineralocorticoid receptor antagonist.  ^a^ Others included alpha-receptor blocker, central antihypertensive drugs.  **^b^***p* values for categorical data generated using the Pearson’s chi-square test for 2 independent proportions. | | | | | | | | | |

| Table S5. Collinearity diagnostics steps | | | | |
| --- | --- | --- | --- | --- |
|  | All indicators | | Not including total cholesterol | |
|  | Tolerance | VIF | Tolerance | VIF |
| Sex | 0.559 | 1.789 | 0.559 | 1.788 |
| Age | 0.56 | 1.786 | 0.561 | 1.783 |
| Married | 0.983 | 1.017 | 0.983 | 1.017 |
| Occupation | 0.845 | 1.184 | 0.845 | 1.184 |
| Current smoker | 0.667 | 1.5 | 0.667 | 1.5 |
| Current drinker | 0.637 | 1.57 | 0.637 | 1.569 |
| Comorbidities |  |  |  |  |
| Ischemic heart disease | 0.844 | 1.184 | 0.845 | 1.184 |
| Chronic cerebral hypoperfusion | 0.937 | 1.067 | 0.937 | 1.067 |
| Chronic kidney diseases | 0.907 | 1.103 | 0.916 | 1.092 |
| Diabetes | 0.887 | 1.128 | 0.887 | 1.128 |
| Duration of hypertension | 0.848 | 1.179 | 0.848 | 1.179 |
| Clinical characteristics |  |  |  |  |
| Systolic BP | 0.748 | 1.337 | 0.748 | 1.337 |
| Diastolic BP | 0.59 | 1.695 | 0.59 | 1.695 |
| Total cholesterol | 0.105 | 9.569 |  |  |
| Triglyceride | 0.3 | 3.33 | 0.878 | 1.14 |
| HDL-cholesterol | 0.532 | 1.881 | 0.802 | 1.247 |
| LDL-cholesterol | 0.143 | 6.975 | 0.931 | 1.074 |
| Uric acid | 0.74 | 1.352 | 0.741 | 1.35 |
| Body mass index | 0.919 | 1.088 | 0.921 | 1.085 |
| *BP* blood pressure; *eGFR* estimated glomerular filtration rate; *LDL-cholesterol* low-density lipoprotein cholesterol; *HDL-cholesterol* high-density lipoprotein cholesterol;*VIF variance inflation factor.* | | | | |

| Table S6. Sensitivity analysis of the relationship between care by hypertension specialists and MACE after sIPTW | | | | | | | |
| --- | --- | --- | --- | --- | --- | --- | --- |
|  | Specialists Group | |  | Non-specialists Group | |  | |
|  | Cases with event, | Incidence rate (cases/ |  | Cases with event, | Incidence rate (cases/ | Adjusted HR ^a^ | |
|  | no. (%) | 1000 person years) |  | no. (%) | 1000 person years) | (95% CI) | *p* value |
| excluding participants with MACE ≤1 year | 381(7.8) | 18.9 |  | 694(15.4) | 34.6 | 0.71(0.59-0.85) | <0.001 |
| (n=9395) |  |  |  |  |  |  |  |
| excluding participants with IHD | 354(7.0) | 19.6 |  | 434(13.3) | 33.7 | 0.69(0.57-0.83) | <0.001 |
| (n=8283) |  |  |  |  |  |  |  |
| excluding participants with CCH | 123(6.3) | 16.9 |  | 460(14.2) | 34.1 | 0.72(0.56-0.91) | 0.007 |
| (n=5184) |  |  |  |  |  |  |  |
| excluding participants with CKD | 400(7.4) | 20.3 |  | 744(15.7) | 38.7 | 0.65(0.54-0.77) | <0.001 |
| (n=10175) |  |  |  |  |  |  |  |
| excluding participants with DM | 304(6.8) | 19 |  | 376(13.8) | 34.3 | 0.62(0.51-0.75) | <0.001 |
| (n=7193) |  |  |  |  |  |  |  |
| Overall | 439(7.8) | 21.5 |  | 806(16.0) | 39.7 | 0.67(0.57-0.79) | <0.001 |
| *MACE* major adverse cardiovascular events; *sIPTW* stabilized inverse probability of treatment weighting; *IHD* ischemic heart diseases;*CCH* Chronic cerebral hypoperfusion; *CKD* chronic kidney diseases; *DM* diabetes mellitus;*CI* confidence interval; *HR* hazard ratio.  ^a^ Adjusted for sex, age, baseline body-mass index, systolic BP, diastolic BP, duration of hypertension, married, occupation, current smoker, current drinker, triglyceride, low-density lipoprotein cholesterol, high-density lipoprotein cholesterol, uric acid and baseline comorbidities. | | | | | | | |

| Table S7. The relationship between care by hypertension specialists and MACE by type of hypertension after sIPTW | | | | |
| --- | --- | --- | --- | --- |
|  | Unadjusted HR |  | Adjusted HR |  |
|  | (95% CI) | *p* value | (95% CI) ^a^ | *p* value |
| Specialists Group |  |  |  |  |
| Essential hypertension | 0.79(0.62-1.02) | 0.070 | 0.67(0.54-0.84) | <0.001 |
| Secondary hypertension | 0.67(0.54-0.84) | <0.001 | 0.67(0.54-0.83) | <0.001 |
| Non-specialists group | Ref. |  | Ref. |  |
| *MACE* major adverse cardiovascular events; *sIPTW* stabilized inverse probability of treatment weighting; *HR* hazard ratio; *CI* confidence interval.  ^a^ Adjusted for sex, age, baseline body-mass index, systolic BP, diastolic BP, duration of hypertension, married, occupation, current smoker, current drinker, triglyceride, low-density lipoprotein cholesterol, high-density lipoprotein cholesterol, uric acid and baseline comorbidities. | | | | |

| Table S8. Subhazards ratio and 95% CI for MACE using a Fine-Gray Proportional Subhazards Model after sIPTW | | | | |
| --- | --- | --- | --- | --- |
|  | Unadjusted sHR |  | Adjusted sHR |  |
|  | (95% CI) | p value | (95% CI)^a^ | p value |
| MACE | 0.56(0.50,0.63) | <0.001 | 0.63(0.55,0.72) | <0.001 |
| *MACE* major adverse cardiovascular events;*sIPTW* stabilized inverse probability of treatment weighting; *sHR* subdistribution hazard ratio; *CI* confidence interval. ^a^ Adjusted for sex, age, baseline body-mass index, systolic BP, diastolic BP, duration of hypertension, married, occupation, current smoker, current drinker, triglyceride, low-density lipoprotein cholesterol, high-density lipoprotein cholesterol, uric acid and baseline comorbidities. | | | | |

| Table S9. E-values for unmeasured confounding between medical care by hypertension specialists and MACE | |
| --- | --- |
|  | the point estimate the lower bound of the 95%CI |
| MACE | 2.35 1.85 |
| *MACE* major adverse cardiovascular events; *CI* confidence interval. | |
